# Independent factors for the development of vasoplegic syndrome in patients undergoing coronary artery bypass surgery

**DOI:** 10.3389/fcvm.2024.1446861

**Published:** 2024-09-10

**Authors:** Constantin L. Palm, Lukas Baumhove, Simon Pabst, Ulf Guenther, Malte Book, Onise Chaduneli, Andreas Martens, Friedrich Mellert, Oliver Dewald

**Affiliations:** ^1^ Department of Cardiac Surgery, Oldenburg Clinic, University of Oldenburg, Oldenburg, Germany; ^2^ Department of Cardiology, University Medical Center Groningen, University of Groningen, Groningen, Netherlands; ^3^ Department of Anesthesiology, Oldenburg Clinic, University of Oldenburg, Oldenburg, Germany; ^4^ Department of Cardiac Surgery, University Hospital Erlangen, University of Erlangen-Nuremberg, Erlangen, Germany

**Keywords:** vasoplegic syndrome, coronary artery bypass graft surgery, Bretschneider cardioplegia, warm blood cardioplegia, extracorporeal circulation (ECC)

## Abstract

**Objective:**

Vasoplegic syndrome remains a common complication of cardiac surgery. It has serious implications for the healthcare system and individual patients, as it leads to rising healthcare costs and higher mortality. A better understanding of factors triggering vasoplegic syndrome is essential for the development of effective prevention strategies. We aimed to identify clinical characteristics and intraoperative parameters associated with the development of vasoplegic syndrome in coronary artery bypass graft surgery and the influence of vasoplegia on outcome.

**Methods:**

We retrospectively analyzed the data of all patients who underwent isolated coronary artery bypass graft surgery or coronary artery bypass graft surgery combined with atrial appendage occlusion, using the heart-lung machine at our institution from 04/2019 to 12/2020. Vasoplegic syndrome was defined as MAP ≤60 mmHg and norepinephrine equivalence dosage of ≥0.2 μg/kg/min with a central venous saturation ≥60% within 2 days from surgery.

**Results:**

Of 647 patients included in this study, 72 (11.1%) developed vasoplegic syndrome. Patients experiencing vasoplegia had longer stay in ICU, more frequently underwent tracheostomy and suffered more often from pneumonia. The duration of extracorporeal circulation, intraoperative application of platelet concentrates and usage of cold crystalloid cardioplegia (Bretschneider) independently predicted development of vasoplegic syndrome.

**Conclusions:**

Even in relatively low-risk cardiac surgery, vasoplegic syndrome is a common complication and was associated with serious adverse effects. The use of warm blood cardioplegia (Calafiore) seems to be safer than cold crystalloid cardioplegia (Bretschneider) and might be preferable in patients that are vulnerable to the consequences of vasoplegic syndrome.

## Introduction

The vasoplegic syndrome was first identified as a consequence of cardiac surgery in 1996 (1). It occurs in up to 33% of patients undergoing cardiopulmonary bypass, depending on the applied definition and type of procedure (2–4). Vasoplegic syndrome has been shown to be associated with prolonged durations of intensive care unit (ICU) stay and overall hospital stay imposing a substantial economic burden on the healthcare system (5). Moreover, it has serious consequences for the affected patients, as it leads to significantly increased mortality (5). While there is no universally accepted definition, frequent common characteristics to define postoperative vasoplegia are low mean arterial blood pressure associated with high necessity for catecholamines with normal cardiac output (6). The underlying pathophysiology is thought to be mainly driven by a pathogen-free inflammatory response to surgical trauma and exposure of blood to the foreign surfaces of the cardiopulmonary bypass circuit (7, 8). Treatment options range from fluid resuscitation and conventional catecholamine therapy to infusion of methylene blue and hydrocortisone (9–11). Each treatment option has potential risk, therefore there is a persistent need for effective strategies to prevent vasoplegia (12, 13). While predisposition for vasoplegic syndrome has been presumed for several factors (14), the search for truly associated and modifiable risk factors is still ongoing.

In this large retrospective study, we aimed to identify pre- and intraoperative risk factors for vasoplegic syndrome in patients undergoing coronary artery bypass grafting (CABG) and relate them to in-hospital outcome.

## Methods

### Data acquisition

This study was conducted in accordance with the declaration of Helsinki. The institutional ethics committee approved the study and waived the need for individual written patient consent. We retrospectively screened data of patients that underwent isolated coronary-bypass surgery or coronary bypass surgery with atrial appendage occlusion as the only additional procedure between 04/2019 and 12/2020 in the Department of Cardiac Surgery at the Oldenburg Clinic in Oldenburg, Germany. The inclusion criteria comprised isolated CABG or CABG with atrial appendage occlusion, use of the heart-lung machine, and induction of cardiac arrest via cardioplegic solution. All operations were performed using venous two-stage cannulation. Anesthesia was induced with propofol, sufentanil, and a non-depolarizing muscle relaxant. The maintenance of anesthesia was conducted using isoflurane and sufentanil. The perioperative antibiotic prophylaxis regimen comprised the administration of 2 g of cephazolin at three time points: induction of anesthesia, immediately following weaning from extracorporeal circulation, and 6 h post-weaning. Blood lost intraoperatively was routinely collected in the cell saver reservoir, further processing and reinfusion was performed if a volume >500 ml was collected. The activated clotting time target was >400 s, the standard heparin dose 500 IE/kg. Cardiac arrest was induced by antegrade infusion of either Bretschneider or Calafiore solution, chosen at the discretion of the operating surgeon. Patients were excluded if ICU data were incomplete, if they had an ejection fraction of less than 30% before or up to 2 days after the operation, or if they underwent re-sternotomy within 2 days after the index surgery. Furthermore, patients that developed cardiogenic shock requiring mechanical support such as extracorporeal membrane oxygenation, intra-aortic balloon pump or left ventricular assist device were excluded. Patients who were administered oral or intravenous immunosuppression excluded. Similarly, patients who had been receiving oral or intravenous antibiotics for reasons other than standard perioperative prophylaxis were also excluded. A complete overview of the inclusion and exclusion criteria is provided in Supplementary Table 1.

The obtained data included demographic data, medical history, preoperative laboratory values, intraoperative surgical data and postoperative data during the ICU stay. Furthermore, medication used within 24 h preoperatively was documented. Outcome parameters were restricted to intrahospital parameters. For a detailed list of the outcome definitions, please refer to Supplementary Table 2.

### Definition of vasoplegic syndrome

We defined patients as vasoplegic in cases when they had in at least one postoperative episode a mean arterial pressure (MAP) ≤60 mmHg for longer than 30 min despite appropriate management of hypotension, a noradrenaline equivalence (NE) dosage of ≥0.2 μg/kg/min, and a central venous saturation of ≥60% within 2 days after surgery. The noradrenaline equivalence (NE) dosage was calculated using the formula of Goradia et al. (15): NE = norepinephrine + epinephrine + phenylephrine/10 + dopamine/100 + metaraminol/8 + vasopressin × 2.5 + angiotensin II × 10.

### Statistical analysis

Statistical analyses were carried out with STATA 17.0 software. Normally distributed data are presented as mean ± standard deviation, non-normally distributed data are displayed as median with interquartile ranges. Baseline characteristics are presented comparing vasoplegic and non-vasoplegic patients. Continuous data were compared using either two-sided *t*-test or Mann-Whitney *U* test where appropriate. Categorical data were compared using Chi-squared test or Fishers exact test depending on the expected number of observations. For identification of independent predictors of vasoplegia, a multivariable forward stepwise approach with a cutoff of *p *< 0.1 was chosen. To correct for clinical confounders, age, sex, selected preoperative medication and comorbities were forced in the analyses. To investigate potential associations between the volume of applied cardioplegia and the development of vasoplegia, separate univariable logistic regressions for volume of applied cardioplegia were carried out for each cardioplegic solution. Further, interaction terms between the use of Bretschneider cardioplegia and age, sex, ACEi use, hypertension, diabetes type II (DM II), eGFR, KoronarCHirurgie Score (KCH-Score), logarithmic EuroSCORE, additive EuroSCORE and Troponin T were included. For this purpose, continuous variables were transformed into categorical variables in below or above the median.

## Results

In total 647 patients were included, of which 72 (11.1%) developed vasoplegia. Demographics, medical history including medication, preoperative risk scores and selected laboratory tests are displayed in Table 1. In the vasoplegia group, angiotension-converting enzyme inhibitors (ACEi) were more frequently used (27.8% vs. 17.9%, *p *= 0.045). Vasoplegic patients had significantly higher additive (5.5 (3.0, 8.0) vs. 4.0 (2.0, 6.0), *p *= 0.016) and logarithmic (4.24 (2.3, 9.1) vs. 3.3 (1.7, 6.1), *p* = 0.023) EuroSCORE than non-vasoplegic patients. The KCH-Score was also higher in vasoplegic than non-vasoplegic patients (1.58 (0.7, 3.0) vs. 1.13 (0.6, 1.9), *p *= 0.011). The preoperative laboratory tests showed higher levels of high-sensitive troponin T values in vasoplegic than in non-vasoplegic patients (26 ng/L (12, 362) vs. 17 ng/L (9, 47), *p *= 0.026). A comprehensive overview of laboratory tests is displayed in Supplementary Table 3.

**Table 1:** Baseline characteristics of non-vasoplegic and vasoplegic patients.

Variables	Level	Non-vasoplegia (*N* = 575)	Vasoplegia (*N * = 72)	*p*-value
Demographics				
Age (years)		66 (9)	68 (8)	0.17
Female sex		101 (17.6%)	17 (23.6%)	0.21
BMI (kg/m^2^)		28.6 (4.4)	28.7 (4.1)	0.80
Surgical urgency	ELective	118 (20.7%)	11 (15.3%)	0.28
	Urgent	413 (72.5%)	53 (73.6%)	
	Emergency	39 (6.8%)	8 (11.1%)	
Medical history				
Atrial fibrillation	Paroxysmal	35 (6.1%)	1 (1.4%)	0.12
	Permanent	32 (5.6%)	5 (6.9%)	
	Persistent	4 (0.7%)	2 (2.8%)	
Arterial hypertension		508 (88.3%)	63 (87.5%)	0.83
Previous CPR	<4 weeks	6 (1. 0%)	2 (2.8%)	0.31
	>4 weeks	6 (1.0%)	0 (0.0%)	
Prior myocardial infarction	<4 weeks	198 (34.4%)	31 (43.1%)	0.34
	>4 weeks	103 (17. 9%)	12 (16.7%)	
Diabetes mellitus type II		199 (34.6%)	24 (33.3%)	0.58
Peripheral artery disease		68 (11.8%)	9 (12.5%)	0.87
Prior stroke		56 (9.7%)	12 (16.7%)	0.071
COPD		58 (10.1%)	9 (12.5%)	0.53
Chronic kidney disease		58 (10.1%)	6 (8.3%)	0.64
NYHA class	I	67 (11.7%)	12 (16.7%)	0.25
	II	198 (34.4%)	17 (23.6%)	
	III	201 (35.0%)	30 (41.7%)	
	IV	8 (1.4%)	2 (2.8%)	
Preoperative LVEF <50%		110 (19.4%)	19 (27.1%)	0.13
**Additive euroSCORE (%)**	** **	**4** **(2, 6)**	**5.5** **(3, 8)**	**0** **.** **016**
**Logarithmic euroSCORE (%)**		**3.3** **(1.7, 6.1)**	**4.24** **(2.3, 9.1)**	**0** **.** **023**
**KoronarCHirurgie score (%)**		**1.13** **(0.6, 1.9)**	**1.58** **(0.7, 3)**	**0** **.** **011**
Medication				
**ACEi**		**103** (**17.9%)**	**20** (**27.8%)**	**0**.**045**
Aldosteronantagonist		14 (2.4%)	3 (4.2%)	0.39
Betablocker		330 (57.4%)	40 (55.6%)	0.77
Calcium antagonist		106 (18.4%)	19 (26.4%)	0.11
Intravenous heparin		192 (33.9%)	23 (31.9%)	0.74
Intravenous nitrate		48 (8.3%)	7 (9.7%)	0.69
Sartane		93 (16.2%)	12 (16.7%)	0.91
Preoperative laboratory values				
Hemoglobin (g/dl)		14.1 (12.9, 15)	13.7 (12.9, 14.8)	0.24
Creatinine (mg/dl)		0.9 (0.8, 1.1)	0.9 (0.8, 1.1)	0.15
**Troponin T (ng/L)**		**17** (**9, 47)**	**26** (**12, 362)**	**0**.**026**

Continuous data are presented as mean (SD) or median (IQR). Categorical variables are displayed as numbers (%). BMI, body mass index; CPR, cardiopulmonary resuscitation; COPD, chronic obstructive pulmonary disease; NYHA, New York Heart Association; LVEF, left ventricular ejection fraction; ACEi, angiotensin-converting enzyme inhibitor. Statistically significant differences are indicated in bold.

**Figure 1:**
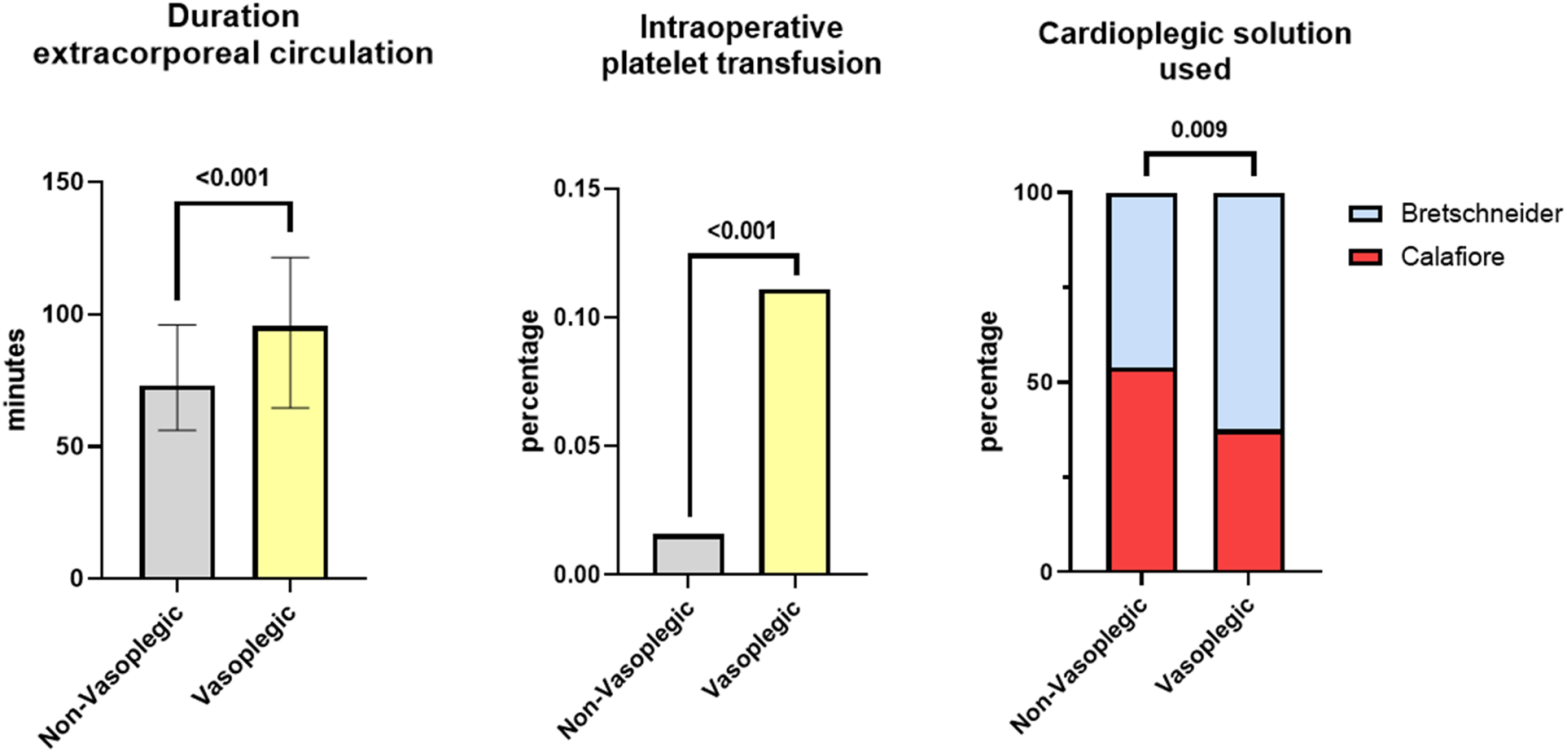
Distribution of independent predictors of vasoplegic syndrom in non-vasoplegic and vasoplegic patients.

Vasoplegic patients experienced significantly longer extracorporeal circulation times (96 min (65, 122) vs. 73 min (56, 57), *p *< 0.001) as well as longer ischemia times (51 min (34, 73) vs. 42 min (30, 57) *p *= 0.002). Moreover, intraoperative administration of platelet concentrates) was more frequent in vasoplegic than non-vasoplegic patients (11.1% vs. 1.6%, *p *< 0.001). With regard to cardioplegic solution for intraoperative cardiac arrest, a significantly higher rate of Bretschneider cardioplegic solution was applied in the vasoplegic group than in non-vasoplegic group (62.5% vs. 46.1%, *p = *0.009, Figure 1). At the same time, higher volumes of Bretschneider solution were used in the vasoplegic group when compared to the non-vasoplegic group (1,044 ± 337 ml vs. 917 ± 272 ml, *p *= 0.006). Among patients receiving Calafiore cardioplegia, the volume of applied cardioplegic solution did not differ significantly (827 ± 306 ml vs. 746 ± 254 ml, *p *= 0.12). Intraoperative characteristics are presented in Table 2 and Figure 1. The results of the univariable logistic regression are presented in Supplementary Table 4.

**Table 2:** Intraoperative characteristics of non-vasoplegic and vasoplegic patients.

Variable	Level	Non-vasoplegic (*N* = 575)	Vasoplegic (*N* = 72)	*p*-value
Redo surgery		6 (1.0%)	2 (2.8%)	0.21
Type of procedure	Isolated CABG	528 (91.8%)	67 (93.1%)	0.72
	CABG + rhythm surgery	47 (8.2%)	5 (6.9%)	
Heart-lung machine details				
**ECC duration (min)**		**73** (**56, 96)**	**96** (**65, 122)**	**<0**.**001**
**Ischemia duration (min)**		**42** (**30, 57)**	**51** (**34, 73)**	**0**.**002**
Priming volume (ml)		1,500 (1,500, 1,500)	1,500 (1,500, 1,500)	0.84
Cardioplegia details				
**Cardioplegia solution**	**Bretschneider**	**265** (**46.1%)**	**45** (**62.5%)**	**0**.**009**
** **	**Calafiore**	**310** (**53.9%)**	**27** (**37.5%)**	
**Cardioplegia volume per type of Cardioplegia (ml)**	**Bretschneider**	**917** (**272)**	**1,044** (**337)**	**0**.**006**
** **	Calafiore	746 (254)	827 (306)	0.12
Number of cardioplegia applications per type of cardioplegia	Bretschneider	1 (1, 1)	1 (1, 1)	0.14
** **	Calafiore	2 (1, 3)	3 (1, 5)	0.14
Volume management				
Transfusion RBCs intraoperative		114 (19.9%)	20 (27.8%)	0.12
**Transfusion FFP intraoperative**		**7** (**1.2%)**	**5** (**6.9%)**	**<0**.**001**
**Transfusion platelet concentrate intraoperative**		**9** (**1.6%)**	**8** (**11.1%)**	**<0**.**001**
Volume cell saver intraoperative (ml)	** **	0 (0, 400)	0 (0, 400)	0.40
Volume crystalloids intraoperative (ml)		3,000 (2,500, 3,500)	3,000 (2,500, 3,500)	0.32

Continuous data are presented as mean (SD) or median (IQR). Categorical variables are displayed as numbers (%). CABG, coronary artery bypass graft surgery; ECC, extracorporeal circulation; FFP, fresh frozen plasma concentrate. Statistically significant differences are indicated in bold.

In multivariable logistic regression (see Table 3, unadjusted model), extracorporeal circulation (OR 1.19, 95% CI 1.10–1.29), intraoperative platelet concentrate application (OR 7.44, 95% CI 2.07–26.70) and use of Bretschneider cardioplegia (OR 1.88, 95% CI 1.40–3.39) were identified as independent predictors for vasoplegia. These remained significant even after correction for possible clinical confounders, namely age and sex (see Table 3, Model 1), preoperative medication (Table 3, Model 2) and comorbities (Table 3, Model 3). Differences of independent predictors of vasoplegia are visualized in Figure 1. Separate univariable logistic regression for each cardioplegia type showed a significant association of higher cardioplegia volume with vasoplegia in patients receiving Bretschneider solution (see Table 4. OR 1.13, 95% CI 1.03–1.25, *p *= 0.011), but not in patients receiving Calafiore.

**Table 3:** Multivariable logistic analyses of factors associated with the development of vasoplegic syndrome.

	Unadjusted model			Model 1			Model 2			Model 3		
Variable	OR	95% CI	*p*-value	OR	95% CI	*p*-value	OR	95% CI	*p*-value	OR	95% CI	*p*-value
**Bretschneider Cardioplegia**	**1**.**88**	**1.04–3.39**	**0**.**035**	**2**.**34**	**1.37–4.01**	**0**.**002**	**2**.**29**	**1.33–3.94**	**0**.**003**	**2**.**31**	**1.34–3.97**	**0**.**003**
**Platelet concentrate intraoperative**	**7**.**44**	**2.07–26.69**	**0**.**002**	**6**.**65**	**2.21–20.00**	**0**.**001**	**6**.**52**	**2.18–19.57**	**0**.**001**	**6**.**38**	**2.12–19.24**	**0**.**001**
**Duration ECC/10 min**	**1**.**19**	**1.10–1.29**	**<0**.**001**	**1**.**17**	**1.09–1.26**	**<0**.**001**	**1**.**17**	**1.09–1.26**	**<0**.**001**	**1**.**17**	**1.08–1.26**	**<0**.**001**

Model 1: adjusted for age + sex. Model 2: adjusted for age + sex + ACEi use + betablocker use + aldosteronantagonist use + heparin i.v. use. Model 3: adjusted for age + sex + ACEi use + betablocker use + aldosteronantagonist use + heparin i.v use + hypertension + diabetes type II + previous myocardial infarction. ACEi, angiotensin-converting enzyme inhibitor; ECC, extracorporeal circulation. Statistically significant associations are indicated in bold.

**Table 4:** Univariable logistic analyses of influence of volume of cardioplegic solution per type of cardioplegia on the development of vasoplegic syndrome.

Variable	OR	95% Confidence interval		*p*-value
**Volume Bretschneider/100 ml**	**1**.**13**	**1**.**03**	**1**.**25**	**0**.**011**
Volume Calafiore /100** **ml	1.12	0.97	1.28	0.122

Statistically significant associations are indicated in bold.

**Figure 2:**
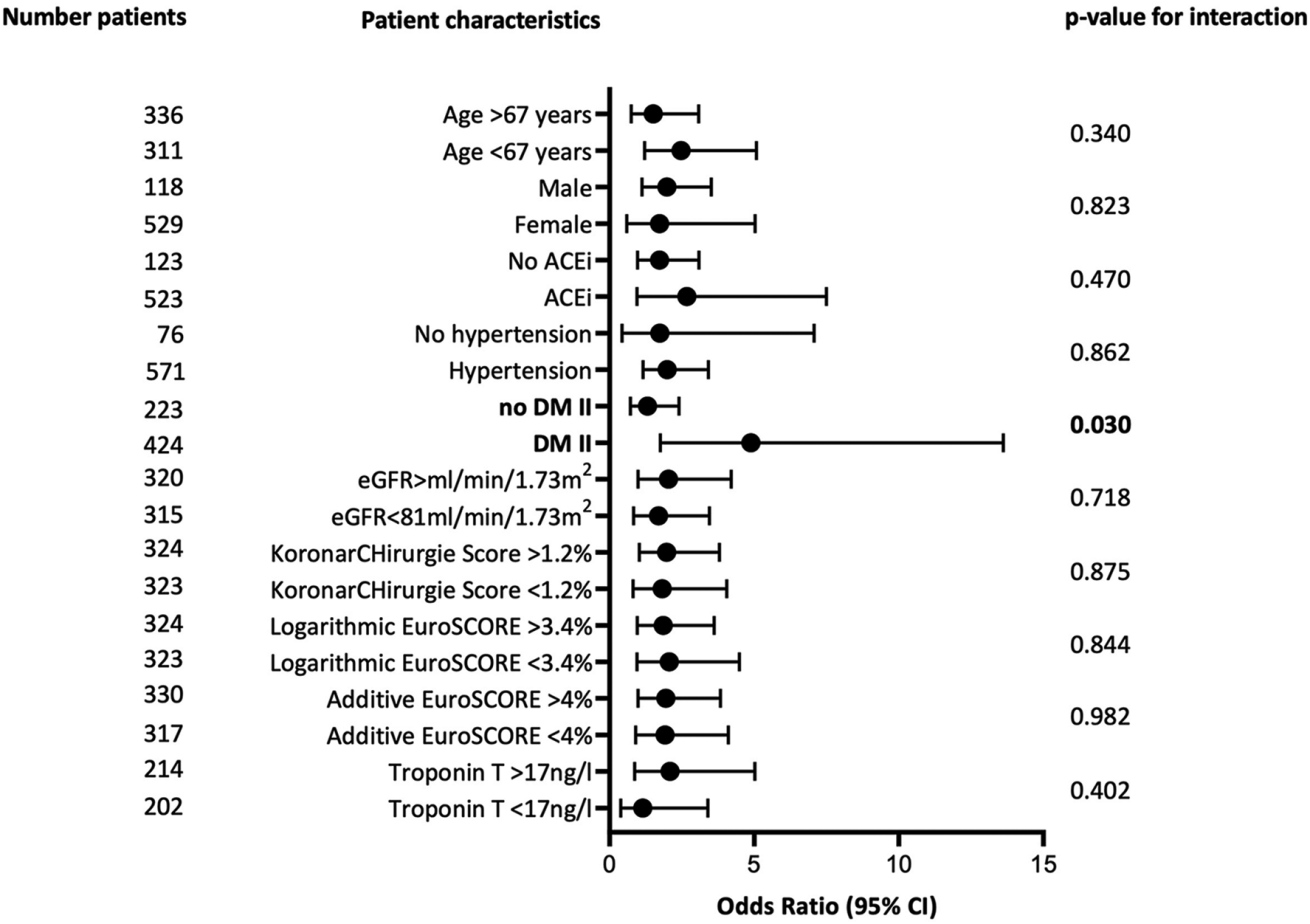
Odds ratios of logistic regression of use of bretschneider cardioplegia on development of vasoplegic syndrome, by relevant patient-level characteristics. ACEi, angiotension-converting enzyme inhibitors. DM II, diabetes type II; eGFR, estimated glomerular filtration rate. Statistically significant interactions are indicated in bold.

Interaction analyses showed a significantly stronger association between the use of Bretschneider cardioplegia and the development of vasoplegia in diabetic patients compared to non-diabetic patients (see Figure 2. OR 4.89, 95% CI 1.76–13.62 vs. 1.31, 95% CI 0.71–2.4, *p for interaction *= 0.030).

Patients who developed vasoplegia had extended length of stay in ICU (4 days (3, 6) vs. 2 days (2, 4) *p *< 0.001) and required longer mechanical ventilation support (17.9 h (13.6, 22.8) vs. 13.3 h (10.5, 16.2), *p *< 0.001). Patients with vasoplegia underwent more frequently tracheostomy (4.2% vs. 0.7%, *p *= 0.007), received more often blood products such as red blood cell concentrates (RBCs; 69.4% vs. 29.2%, *p *< 0.001) and fresh frozen plasma (FFP; 56.9% vs. 22.1%, *p *< 0.001) and exhibited an increased requirement for fluid substitution (4,666 ± 1,995 ml vs. 2,867 ml ± 2,730 ml, *p *< 0.001). Vasoplegic patients developed more often pneumonia than non-vasoplegic patients (8.3% vs., 3.0% *p *= 0.02). Though patients with vasoplegia stayed significantly longer in hospital (11 days (9, 14) vs. 9 days (9, 12), *p *= 0.007), no differences were observed in intrahospital mortality (1.4% vs. 0.5%, *p *= 0.38). Detailed ICU parameters and intrahospital outcomes are in displayed in Table 5.

**Table 5:** ICU stay characteristics and intrahospital outcomes of non-vasoplegic and vasoplegic patients.

Variable	Non-vasoplegic (*N* = 575)	Vasoplegic (*N* = 72)	*p*-value
**Duration ICU stay (days)**	**2** (**2, 4)**	**4** (**3, 6)**	**<0**.**001**
**Total ventilation time (hours)**	**13.25** (**10.5, 16.2)**	**17.88** (**13.6, 22.8)**	**<0**.**001**
**Duration hospital stay (days)**	**9** (**8, 12)**	**11** (**9, 14)**	**0**.**007**
Postoperative LVEF <50%	120 (26.0%)	22 (36.7%)	0.082
Volume management			
**Transfusion RBC**	**168** (**29.2%)**	**50** (**69.4%)**	**<0**.**001**
**Transfusion FFP**	**127** (**22.1%)**	**41** (**56.9%)**	**<0**.**001**
Transfusion platelet concentrate	27 (4.7%)	6 (8.3%)	0.19
**Fluid balance 48 h (ml)**	**+2,867** (**1,995)**	**+4,666** (**2,730)**	**<0**.**001**
Intrahospital outcomes			
Intrahospital cardiac arrest	5 (0.9%)	2 (2.8%)	0.14
Tachyarrhythmia absoluta	154 (26.8%)	17 (23.6%)	0.57
Stroke	12 (2.1%)	3 (4.2%)	0.27
Delirium	52 (9.1%)	9 (12.5%)	0.35
Reintubation	5 (0.9%)	2 (2.8%)	0.14
**Tracheotomy**	**4** (**0.7%)**	**3** (**4.2%)**	**0**.**007**
Pleural effusion requiring drainage	22 (3.8%)	2 (2.8%)	0.66
**Pneumonia**	**17** (**3.0%)**	**6** (**8.3%)**	**0**.**020**
Sepsis	4 (0.7%)	0 (0.0%)	0.48
Dialysis	3 (0.5%)	0 (0.0%)	0.54
Wound healing defects	17 (3.0%)	4 (5.6%)	0.24
HIT II	6 (1.0%)	0 (0.0%)	0.38
Urinary tract infection	15 (2.6%)	0 (0.0%)	0.17
Intrahospital mortality	3 (0.5%)	1 (1.4%)	0.38

Continuous data are presented as mean (SD) or median (IQR). Categorical variables are displayed as numbers (%). ICU, intensive care unit; RBC, red blood cell concentrate; FFP, fresh frozen plasma concentrate; HIT, heparin-induced thrombocytopenia. Statistically significant differences are indicated in bold.

## Discussion

With an incidence of 11.1%, vasoplegia proved to be a prevalent entity associated with serious adverse effects, even in this comparatively low-risk patient cohort undergoing isolated CABG surgery. We identified duration of extracorporeal circulation, intraoperative platelet concentrate transfusion and use of Bretschneider cardioplegia as independent predictors of vasoplegic syndrome.

The incidence of vasoplegia in cardiac surgery is described to be between 7% and 33% (2–4, 16–18), with variations observed across different patient populations. The observed incidence of 11.1% in this study is being consistent with that of previous reports on CABG surgery (16, 18).

Vasoplegic patients preoperatively more often took ACEis (Table 1). From a pathophysiological point of view, preoperative ACEi use might intuitively be considered a risk factor for postoperative vasoplegia as it implies inhibition of a key regulator of blood pressure, the renin-angiotensin-aldosterone system. ACEi use has been described as a risk factor for vasoplegic syndrome in prior studies (17, 19, 20), but although ACEi use was significantly more common in patients with vasoplegia, it did not prove to be predictive of vasoplegia after adjustment in our multivariable analysis (Table 3).

The higher preoperative risk scores, logarithmic, additive EUROScore and KCh score, observed in the vasoplegia group may reflect the increased vulnerability of these patients with impaired overall health to a wider spectrum of complications after cardiac surgery than just mortality. Levin et al. previously described an association between elevated EUROScores and vasoplegic syndrome (2). However, in multivariable analyses, these scores did not show to be independent predictors of vasoplegia. Increased transfusion rates have been previously described to be associated with vasoplegia (21), and our data confirmed this for both perioperative FFP and platelet concentrate transfusion. Moreover, in multivariable analyses, platelet concentrate transfusion was identified as an independent predictor of vasoplegic syndrome. While transfusions have been suspected to cause an inflammatory response and thereby potentially contribute to the development of vasoplegia, we assume a reverse causal relationship where transfusion and volume substitution emerge as a consequence of vasoplegia (22, 23).

Often described risk factors for vasoplegia are duration of surgery and extracorporeal circulation, as well as longer cross-clamp time (4, 24). The relationship between these three parameters and the development of vasoplegic syndrome forms the basis for the common theory that prolonged contact of the blood with the foreign surfaces of the extracorporeal circulation causes a hyperinflammatory state. In 2005, Prondzinsky et al. proposed a higher influence of the surgical trauma than of the cardiopulmonary bypass on inflammatory response (25). Based on this, Dayan et al. proposed, that the increased duration of extracorporeal circulation rather reflects the complexity of surgery and extent of surgical induced trauma (6). Our study had a relatively uniform population in terms of surgical trauma dimensions, as it only included isolated CABG surgery or CABG surgery with atrial appendage occlusion. Nevertheless, longer cross-clamp time and extracorporeal circulation duration were found to be associated with vasoplegia, duration of extracorporeal circulation even showed to be an independent predictor of vasoplegia in our study. These findings are in favor for the hypothesis of a direct effect of cardiopulmonary bypass duration on the development of vasoplegia (26). Members of the heart team should be aware that patients who are expected to undergo longer extracorporeal circulation, for instance due to complex calcification of the coronary arteries or triple vessel disease, are at an elevated risk of developing vasoplegia.

An intriguing finding in our study was the impact of the choice of cardioplegic solution on the development of vasoplegic syndrome, a relationship we are the first to describe. Bretschneider solution was administered significantly more often than blood-based Calafiore in vasoplegic patients. The use of Bretschneider cardioplegia was shown to be an independent predictor of vasoplegia in multivariable analysis and remained significant after adjustment for clinical confounders. The observed effect appears to be volume-dependent, as patients with vasoplegia received higher volumes of Bretschneider solution, and logistic regression analysis showed that higher volumes of Bretschneider solution were associated with the occurrence of vasoplegia. Several mechanisms may underlie this finding. Using Bretschneider cardioplegia leads to a higher infusion of fluid low in sodium and potassium into the body compared to Calafiore (see Supplementary Table 5 for details on Bretschneider composition) (27). The resulting hyperpolarization may inflict vasomotor activity when cardioplegic solution enters the systemic circulation. Activation of calcium channels is pivotal in the induction of vasoconstriction, as high intracellular levels of calcium induce contraction of smooth muscle (7). Voltage-sensitive calcium channels are deactivated by hyperpolarization, leading to inhibition of vasoconstriction (28). The low calcium content of Bretschneider solution may aggravate this effect, as the resulting concentration gradient drives calcium efflux from intracellular to extracellular, thus inhibits vasoconstriction of smooth muscle cells by calcium depletion. Bretschneider cardioplegia could also contribute directly to an inflammatory response as it contains the buffer histidine. Histidine is rapidly metabolized to histamine, a central stimulator of acute inflammation (29). Histamine levels have been shown to be elevated after application of Bretschneider solution (30). Furthermore, Bretschneider cardioplegia has been described to cause hemodilution, which could increase the frequency of transfusions, again causing an inflammatory response to the application of blood products (23, 31). A temporary drop in MAP after induction of cardioplegia by Bretschneider solution has been described previously (30). It is conceivable that this transient phenomenon, if it occurs simultaneously with other aforementioned triggers, increases the risk of vasoplegia in certain patients. The results of the interaction analyses indicated that the association between the use of Bretschneider and the development of vasoplegia is particularly pronounced in patients with DM II. This phenomenon may be due to impaired vasomotor regulation and chronic low-grade inflammation commonly found in diabetic patients. This may predispose diabetic patients to the adverse effects of Bretschneider solution (32, 33).

The findings of this study suggest several possible preventive measures. Given that the vasoplegic effect of Bretschneider was shown to be volume-dependent, strategies aimed at minimising the volume introduced into the systemic circulation during CPB could potentially decrease the occurrence of vasoplegia in cardiac surgery. One straightforward intervention would be to use only the minimal volume necessary to induce cardioplegia. Furthermore, cannulation of both the inferior and superior vena cava, along with separate suction of the cardioplegia solution during induction of cardioplegia, could be protective of vasoplegia. This approach would lead to a reduction in the systemic spill of cardioplegic solution compared to the use of a two-stage cannula, as employed in the present study. Alternatively, retrograde infusion of cardioplegic solution with suction of the aortic root could ensure a reduction in the volume of cardioplegia entering the circulation, however this would limit right ventricular myocardial protection (34, 35). Our data indicate a potential association between the use of Bretschneider and the development of vasoplegia, particularly in patients with diabetes. Consequently, it may be beneficial to consider the avoidance of Bretschneider and the alternative use of Calafiore in diabetic patients undergoing coronary artery bypass graft (CABG) surgery.

The existing literature on the association between vasoplegia and mortality remains inconclusive. While some studies report an elevated risk of mortality of up to 25%, others describe no significant association (1, 20). In this study intrahospital mortality did not differ between vasoplegic and non-vasoplegic patients. This may be due to the selection of a relatively low-risk population for this study, as reflected by a low overall mortality rate. Nevertheless, vasoplegia was found to be associated with serious adverse effects. Vasoplegic patients more frequently developed pneumonia, more often underwent tracheotomy and needed a longer mechanical ventilation support. The resulting increase in ICU and overall hospital stay represent a significant burden for the healthcare system.

Limitations of our study include the retrospective design which means that our findings are based on associations. Results of this study cannot provide proof of causality and should therefore be considered hypothesis-generating. Still, our data suggest that the choice of the applied cardioplegic technique is a modifiable risk factor of vasoplegic syndrome. Therefore, the use of Calafiore cardioplegia might be part of a prevention strategy to reduce the incidence of vasoplegia in cardiac surgery. Although the selection of the vasoplegic solution by the operating surgeon introduces potential bias, the choice of cardioplegic solution was not confined to a single type per surgeon. Surgeons utilized both types of cardioplegic solutions, rather than consistently adhering to a single type. Also, the single-center design may limit the strength of our study. Confirmation of our findings in a prospective approach is warranted. A randomized controlled trial examining the impact of the choice of cardioplegic solution on the incidence of vasoplegia would be particularly valuable. In addition, due to the selection of low-risk patients for inclusion in our study, the generalizability of our findings is limited. The number of redo surgeries is relatively low, which may limit the ability to draw conclusions about the association between redo surgery and the development of vasoplegia. Another potential limitation is that we did not take cardiac index or systemic vascular resistance into account for our definition of vasoplegia. This is due to the restrictive use of Swan-Ganz catheters in low-risk patients at our institution. However, we excluded patients with impaired pre- or post-operative LVEF or those who required mechanical circulatory support, to avoid low-output syndrome as a cause of reduced MAP.

In summary, the present study contributes to a better understanding of this complex syndrome and may help in the development of prevention strategies by identifying several risk factors for vasoplegia. Further refinement and studies on associated factors are needed for effective prevention and treatment of vasoplegic syndrome in cardiac surgery and other medical fields.

## Data Availability

The datasets presented in this article are not readily available because of privacy reasons. Requests to access the datasets should be directed to oliver.dewald@uk-erlangen.de.
