# Supplementary material for: Independent factors for the development of vasoplegic syndrome in patients undergoing coronary artery bypass surgery
Source: Front Cardiovasc Med. 2024 Sep 10;11:1446861. doi: 10.3389/fcvm.2024.1446861 (PMC11420007; doi:10.3389/fcvm.2024.1446861)
Supplement: Supplementary file 1 [file Datasheet1.pdf]

Supplementary material: Independent factors for the development of vasoplegic syndrome in patients undergoing coronary artery bypass surgery

Inclusion criteria	Exclusion criteria
Isolated CABG or CABG plus atrial appendage occlusion for coronary artery disease	Ejection fraction <30 % preoperatively or in the first two days postoperatively
Use of the heart-lung machine	Necessity for extra-corporal mechanical support (ECMO, IABP, Impella)
Application of cardioplegia	Resternotomy within two days after ICU admission
	Oral or intravenous antibiotics for reasons other than standard perioperative prophylaxis
	Oral or intravenous immunosuppression
	Incomplete hemodynamic ICU documentation

Supplementary Table 1: Inclusion and exclusion criteria. CABG= coronary artery bypass graft surgery; ECMO= extracorporeal membrane oxygenation; IABP= intraaortal balloon pump; ICU= intensive care unit.

Intrahospital cardiac arrest: documented intrahospital cardiac arrested requiring cardiopulmonary resuscitation
Tachyarrhythmia absoluta: documented tachycardia with differing R-R-intervals, combined with absence of physiological P-waves
Stroke: documented neurological deficit due to confirmed either ischemic or hemorrhagic central nervous event
Delirium: documented delirium based on the Confusion Assessment Method for Intensive Care Unit (CAM-ICU)
Reintubation: documented endotracheal intubation after postoperative extubation
Tracheotomy: documented tracheotomy
Pleural effusion requiring drainage: documented pleural effusion by radiography, requiring drainage via chest tube insertion
Pneumonia: documented diagnosis of pneumonia diagnosed by clinical assessment and radiographic confirmation of infiltration >48 h after the index surgery
Sepsis: documented sepsis >48 h after the index surgery, diagnosed by a Sequential Organ Failure Score (SOFA) of >2 and detection of a pathogen in blood cultures
Dialysis: documented execution of blood-dialyses
Wound healing defects: documented clinical insufficient wound healing, requiring advanced care
HIT II: documented heparin induced thrombocytopenia type II confirmed by detection of platelet factor 4/heparin antibodies
Urinary tract infection: documented urinary tract infection by clinical diagnosis
Intrahospital mortality: documented intrahospital death

Supplementary Table 2: Definitions of intrahospital outcome parameters.

**Variable**	**Non-Vasoplegic** (N=575)	**Vasoplegic** (N=72)	**p-value**
Leukocytes (10^3/µL)	7.88 (2.13)	8.19 (2.36)	0.26
Platelets (10^3/µL)	250.66 (75.52)	248.69 (70.94)	0.83
Hemoglobin (g/dl)	14.1 (12.9, 15)	13.7 (12.9, 14.8)	0.24
Hematocrit (%)	42 (39, 45)	42 (39, 44)	0.48
Erythrocytes (10^6/µL)	4.7 (4.3, 4.9)	4.6 (4.3, 4.9)	0.47
INR	1 (1, 1.1)	1 (1, 1.1)	0.50
aPTT (sec)	29 (27, 33)	29 (27, 34)	0.56
ATIII (%)	95 (87, 104)	89 (83, 102)	0.084
Creatinin (mg/dl)	0.9 (0.8, 1.1)	0.9 (0.8, 1.1)	0.15
eGFR (ml/min/1.73m^2)	81 (66, 92)	81.5 (67, 95)	0.65
Urea (mg/dl)	31 (25, 39)	33 (27, 43)	0.53
Bilirubin (mg/dl)	0.5 (0.4, 0.7)	0.4 (0.3, 0.6)	0.23
AST (U/l)	26 (21, 34)	26 (21, 42)	0.39
ALT (U/l)	27 (18, 39)	23 (19, 37)	0.47
ChE (kU/l)	8.25 (1.68)	7.80 (1.57)	0.050
LDH (U/l)	193 (169, 227)	208 (174, 235)	0.15
CK (U/l)	82.5 (59, 124)	91.0 (64, 124)	0.35

CKMB mass (U/l)	19 (14, 27)	20 (15.5, 32)	0.26
TSH (mU/l)	1.44 (0.90, 2.22)	1.39 (0.81, 1.77)	0.28
CRP (mg/dl)	0.4 (0.4, 0.7)	0.4 (0.4, 1.1)	0.24
**Troponin T (ng/l)**	**17 (9, 47)**	**26 (12, 362)**	**0.026**
HBsAG positive	2 (0.4 %)	1 (1.6 %)	0.22
antiHBc positive	10 (2.0 %)	1 (1.6 %)	0.83
antiHCV positive	4 (1.6 %)	0 (0.0 %)	0.53

Supplementary Table 3: Preoperative laboratory values of non-vasoplegic and vasoplegic patients. Continuous data are presented as mean (SD) or median (IQR).

Variable		Odds ratio	95% confidence interval		p-value
Age		1.02	0.99	1.05	0.172
Female Sex		0.69	0.38	0.38	0.212
BMI		1.01	0.95	1.06	0.801
Urgency	urgent	1.38	0.70	2.72	0.358
	emergency	2.20	0.83	0.59	0.115
Atrial fibrillation	paroxysmal	0.23	0.03	1.67	0.145
	permanent	1.23	0.46	3.27	0.678
	persistent	3.94	0.71	21.93	0.118
Arterial hypertension		0.92	0.44	1.94	0.833
smoking	active	0.71	0.35	1.42	0.334
	former	0.93	0.54	1.59	0.781
CPR <4weeks ago		2.68	0.53	13.54	0.233
MI <4weeks		1.44	0.88	2.37	0.151
Diabetes mellitus type II		0.95	0.67	1.33	0.755
Peripheral artery disease		1.07	0.51	2.24	0.868
Prior stroke		1.85	0.94	3.65	0.075
COPD		1.27	0.60	2.69	0.527
Chronic kidney disease		0.81	0.34	1.95	0.639
NYHA class	I	1.64	0.69	3.94	0.265
	II	0.79	0.36	1.75	0.558
	III	1.37	0.66	2.85	0.398
	IV	2.30	0.43	12.19	0.329
Preoperative LVEF <50%		1.55	0.88	2.73	0.131
**Additive EuroSCORE**		**1.10**	**1.01**	**1.19**	**0.020**
**Logarithmic EuroSCORE**		1.01	0.99	1.03	0.377
**KoronarCHirurgie Score**		**1.18**	**1.05**	**1.34**	**0.008**
**ACEi**		**1.76**	**1.01**	**3.07**	**0.047**
Aldosteronantagonist		1.74	0.49	6.21	0.392
Betablocker		0.93	0.57	1.52	0.767
Calcium antagonist		1.59	0.90	2.79	0.109
Intravenous heparin		0.91	0.54	1.55	0.738

Intravenous nitrate		1.18	0.51	2.72	0.694
Sartane		1.04	0.54	2.00	0.915
Hemoglobin		0.94	0.81	1.09	0.415
Creatinine		0.41	0.14	1.19	0.102
Troponin T		1.00	1.00	1.00	0.708
Redo surgery		2.71	0.54	13.68	0.228
Type of procedure	CABG + rhythm surgery	0.84	0.32	2.18	0.718
**ECC duration /10min**		**1.16**	**1.08**	**1.25**	**<0.001**
**Ischemia time/10min**		**1.20**	**1.08**	**1.33**	**0.001**
Priming volume		1.00	1.00	1.00	0.966
**Cardioplegia type Bretschneider**		**1.95**	**1.18**	**3.23**	**0.009**
**Transfusion RBCs intraoperative**		**1.54**	**0.89**	**2.70**	**0.122**
**Transfusion FFP intraoperative**		**6.03**	**1.86**	**19.54**	**0.003**
**Transfusion platelet concentrate intraoperative**		**7.83**	**2.92**	**21.01**	**<0.001**
Volume cell saver		1.00	.99	1.01	0.349
Volume crystalloids intraoperative		1.00	.99	1.01	0.307

Supplementary Table 4: Univariable logistic analyses of factors associated with the development of vasoplegic syndrome. BMI= body mass index. COPD= chronic obstructive lung disease. LVEF= left ventricular ejection fraction. ACEi= angiotensin converting enzyme inhibitor. ECC= extracorporeal circulation. RBC= red blood cell concentrate. FFP= fresh frozen plasma. Statistically significant associations are indicated in bold.

	**mmol per 1000 ml**
Sodiumchloride	15
Potassiumchloride	9
Magnesiumchloride-hexahydrate	4
Calciumchloride-dihydrate	0.015
Histidine	180
Histidinehydrochloride Monohydrate	18
Tryptophan	2
Mannitol	30
Potassium-hydrogen-2-ketoglutarate	1

Supplementary Table 5: Composition of Bretschneider cardioplegia in mmol per 1000 ml.
